# Somatosensory ECoG-based brain–machine interface with electrical stimulation on medial forebrain bundle

**DOI:** 10.1007/s13534-022-00256-6

**Published:** 2022-12-20

**Authors:** Yoon Kyung Cho, Chin Su Koh, Youjin Lee, Minkyung Park, Tae Jun Kim, Hyun Ho Jung, Jin Woo Chang, Sang Beom Jun

**Affiliations:** 1 Department of Electronic and Electrical Engineering, Ewha Womans University, 52 Ewhayeodae-gil, Seodaemun-gu, Seoul, 03760 Republic of Korea; 2 Department of Neurosurgery, Yonsei University College of Medicine, 50 Yonsei-ro, Seodaemun-gu, Seoul, Republic of Korea; 3 Graduate Program in Smart Factory, Ewha Womans University, Seoul, South Korea; 4 Brain Korea 21 PLUS Project for Medical Science and Brain Research Institute, Yonsei University College of Medicine, Seoul, Republic of Korea; 5 Division of Brain and Cognitive Sciences, Scranton College, Ewha Womans University, Seoul, Republic of Korea

**Keywords:** Brain–machine interface, Somatosensory cortex, Virtual reward, Brain plasticity, Deep brain stimulation

## Abstract

Brain–machine interface (BMI) provides an alternative route for controlling an external device with one’s intention. For individuals with motor-related disability, the BMI technologies can be used to replace or restore motor functions. Therefore, BMIs for movement restoration generally decode the neural activity from the motor-related brain regions. In this study, however, we designed a BMI system that uses sensory-related neural signals for BMI combined with electrical stimulation for reward. Four-channel electrocorticographic (ECoG) signals were recorded from the whisker-related somatosensory cortex of rats and converted to extract the BMI signals to control the one-dimensional movement of a dot on the screen. At the same time, we used operant conditioning with electrical stimulation on medial forebrain bundle (MFB), which provides a virtual reward to motivate the rat to move the dot towards the desired center region. The BMI task training was performed for 7 days with ECoG recording and MFB stimulation. Animals successfully learned to move the dot location to the desired position using S1BF neural activity. This study successfully demonstrated that it is feasible to utilize the neural signals from the whisker somatosensory cortex for BMI system. In addition, the MFB electrical stimulation is effective for rats to learn the behavioral task for BMI.

## Introduction

Nerve damage or neurodegenerative diseases often lead to a loss of motor or sensory function. In some cases, the impairment is so severe that it does not allow voluntary movement or sensation. Brain–machine interfaces (BMIs) have emerged as practical options to restore the abilities of patients who have lost the motor or sensory function [1–3]. In general, BMIs can decode a user’s intentions from acquired brain signals and then help control external devices to allow the user to interact with external environments. Although the application of BMI is aimed for humans, the animal models with rodent or primate models have also played an essential role in the development of BMI systems. Chapin et al*.* showed that a motor-based BMI rat model can control a lever moving in one dimension to obtain a reward using real-time multichannel spike signals [4]. Based on this work, several BMI systems have been developed for controlling artificial actuators operated by neuronal recordings in the motor cortex, predominantly from the primary motor cortex, but occasionally from the premotor cortex and supplementary motor area [5–11]. Traditionally, the motor cortex has been the main target of BMIs; however, other brain areas including the prefrontal (PFC) or parietal cortex also have the potential for BMI applications [10–18]. The PFC and posterior parietal cortex (PPC) belong to a broader brain network involved in controlling cognitive functions such as working memory, spatial attention, and decision-making [13, 14]. Lang et al. developed a rat model controlling a BMI system using neuron activity in the PFC [15]. Musallam et al. used signals from the PPC of monkeys for controlling cursor position on a computer screen [17]. Jung et al. developed a simple motor cortex-based BMI system and compared its performance between the frontal, motor, and parietal cortexes [18].

Sensory cortex is also often used for BMI system as a stimulation target for restoring sensation and mimicking a sensorimotor loop similar to a somatosensory system’s biofeedback after movement [10, 11, 19]. For example, the studies aiming to develop a BMI system with dexterous robotic upper limb used intracortical microstimulation on the somatosensory cortex to provide artificial tactile feedback to guide BMI control in addition to visual feedback [20, 21]. Apart from processing afferent somatosensory inputs, it was shown that the primary somatosensory cortex is involved in motor planning in various human studies [22, 23]. In the case of rodent animals, the whisker-related primary somatosensory cortex (S1BF) does not only processes sensory information from whisker but also directly influences whisker motor control [24, 25]. Neuron tracing study showed that S1BF projected strongly to the spinal trigeminal nuclei where the whisker-related primary motor cortex apparently projected [24]. It was also reported that there is a projection from S1BF to an ipsilateral whisker-related primary motor cortex [25]. Besides the connectivity to the motor cortex, S1BF has the substantial connectivity with various brain areas, such as the PPC, dorsolateral striatum, posterior medial nucleus, and ventral posterior medial nucleus of the thalamus.

Related with the BMI system, it is important that the information from the whisker somatosensory cortex can be used to determine the intent of the animal. Recently, the researchers showed that S1BF directly controls whisker movements and plays a role to whisker-related learning of goal-directed behaviors [26]. Interestingly, simple whisker-dependent task studies, where animals learned to lick a reward spout in response to a perceived whisker stimulus, have shown that reward-based learning changed the response of cortex [27–29]. Le Merre et al*.* showed that medial prefrontal cortex and dorsal hippocampus responded to the sensory-evoked signals after task learning and correlated with behavioral performance [29]. Therefore, it is expected that the sensory neural activity of a trained animal can be used to control BMI.

Several studies showed that the rats can be trained for specific behaviors via electrical stimulation of brain areas, primarily focusing on the dopaminergic pathway as a target for reward-directed behavior [30–32]. Strongly linked with activation of dopaminergic neurons, the stimulation of the medial forebrain bundle (MFB) induces pleasant bodily sensations, resulting in the delivery of a reward [30]. MFB connects between ventral tegmental area (VTA) and the nucleus accumbens (NAc), which is called mesolimbic dopaminergic pathway. Talwar et al*.* showed rat navigation with MFB stimulation [31], in which rat’s movement and action were indirectly controlled by the virtual rewards. This virtual reward with MFB stimulation strongly motivated the animal to perform as expected to earn more reward.

In this study, we implemented a BMI system with a rat model to explore whether the intention of the animals can be extracted from the somatosensory cortex combined with the MFB electrical stimulation. We trained the animal with virtual reward to control the BMI tasks. Electrocorticographic (ECoG) signals from both the left and right hemispheres of the whisker barrel cortex, which receive input from the whiskers, were recorded, and animals were operant conditioned by electrically stimulating the MFB. The proposed BMI system compared the right and left brain-derived neural signal in a selected gamma band, then a dot on the screen moved horizontally in x-axis under control of animal’s intention. The next position of the dot was decided by comparing the neural signals of the somatosensory cortex. The BMI task training was performed for 7 days, and the animals’ performance improved during this period.

## Materials and methods

### Overview of the proposed BMI system

**Fig. 1:**
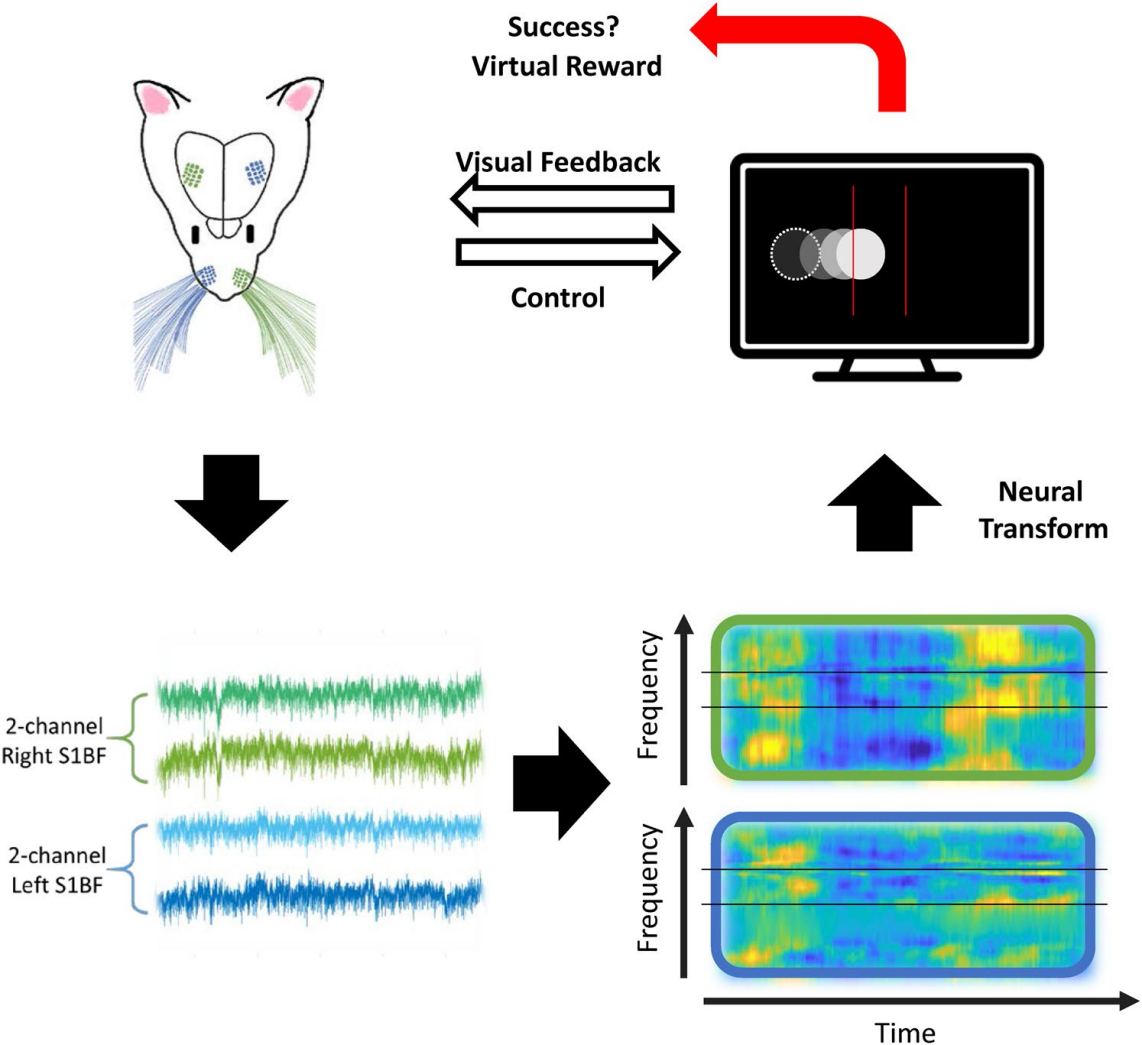
Schematic representation of the BMI system operation. The ECoG signals of the S1BF were collected from a freely moving rat in real-time. The power spectrum of the gamma band (40–70 Hz) in the right (green) and left (blue) barrel cortex were compared and transformed into a dot position on a screen. Simultaneously, the rat watched the screen and tried to control the dot to move it to the screen center. MFB electrical stimulation was provided as a reward for task completion

The BMI system included four components: signal acquisition, electrical stimulation, a controller, and a monitor. An operant-conditioned rat was placed in a chamber where the animal could easily see a white dot on the black screen in a dark experiment room. The white dot appeared at the end of the monitor and the animal tried to control and move it to the center of the screen to earn a virtual reward (Fig. 1). Four-channel ECoG signals from the S1BF were recorded and MFB was stimulated in freely behaving rats. The amplitude spectrum between 40 and 70 Hz of both the right and left hemispheres were compared and the next displacement of the dot was decided based on the direction of the larger amplitude spectrum between the two hemispheres. The BMI task was considered a success when the animal could control the dot position in the screen center. The performance was monitored in terms of the success rate during the 7 day-training period.

### Surgical procedure

Six male Sprague–Dawley rats (300–330 g) were used in this study. Rats were allowed to acclimate for at least 1 week prior to surgery. Animals were housed individually in single laboratory cages with ad libitum access to food and water in a 12-h light/dark (lights on at 8:00 am) cycle with controlled temperature (22 ± 2 °C) and humidity (55 ± 5%). All experimental procedures were conducted in compliance with the Guide for the Care and Use of Laboratory Animals of the National Institutes of Health and approved by the Institutional Animal care and Use Committee of Yonsei University (approval number: 2019-0228). The rats were anesthetized by intraperitoneal injection of a mixture of ketamine (75 mg/kg), acepromazine (0.75 mg/kg), and xylazine (4 mg/kg). After fixing the rat on a stereotaxic frame, the bregma was horizontally aligned to the height of the lambda. Subsequently, we performed a midline scalp incision and cleaned the exposed skull with 3% hydrogen peroxide solution.

**Fig. 2:**
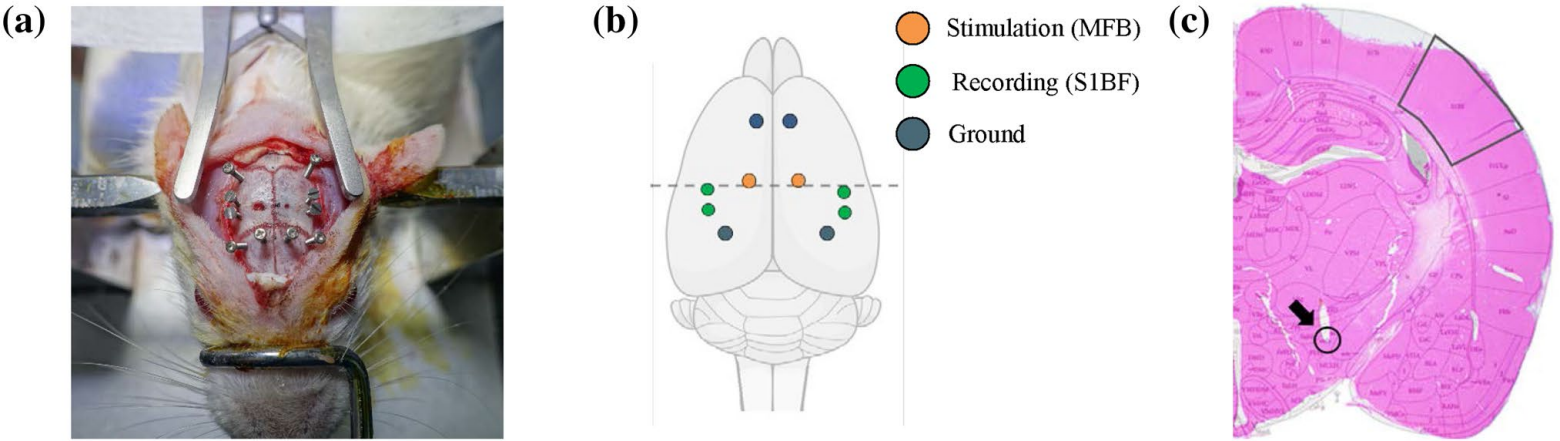
Electrode implantation and position confirmation in the rat brain **a** image acquired during surgery depicting the position of electrodes and anchors. **b** Locations of the implanted electrodes. Dashed line indicates the cross section of **c**. **c** MFB (arrow) and S1BF (box) locations in the coronal brain section

A total of 10 holes were drilled into the skull for electrode implantation (Fig. 2a, b). For MFB stimulation, four bilateral burr holes were drilled into the skull for two MFB electrodes (anteroposterior [AP]: − 2.3 mm, mediolateral [ML]: ± 1.8 mm) and two ground electrodes (AP: + 2.5 mm, ML: ± 1.6 mm) as shown in Fig. 2b. Six bilateral burr holes were drilled for somatosensory signal acquisition. Four holes were for recording electrodes in somatosensory cortex (AP: − 1.5 mm, ML: ± 5.5 mm and AP: − 2.5 mm, ML: ± 5.5 mm) and two holes were for ground electrodes (AP: − 7.5 mm, ML: ± 5.5 mm). For stable recording, neural signal recording was acquired from two channels of each hemisphere. Subsequently, customized tungsten electrodes (diameter: bare 127 μm, coated 178 μm; A-M Systems, LLC., Washington, USA) were stereotactically inserted into the lateral hypothalamus for MFB stimulation (dorsoventral − 8.6 mm) through a burr hole (AP − 2.3 mm, ML ± 1.8 mm) [30]. We used only one electrode to control the rat’s behavior via electrical stimulation, but we implanted electrodes in both hemispheres to increase the success rate of operant conditioning. Stainless steel screws (tip diameter 0.8 mm) were inserted into other burr holes to serve as somatosensory neural signal recording and ground electrodes. Two additional stainless steel screws were inserted near ground as anchors. All electrodes were firmly fixed using dental cement. After the whole experiments, the rats were sacrificed and the brains were fixed, sectioned, and stained with hematoxylin and eosin (H&E) to confirm electrode positions (Fig. 2c).

### MFB stimulation parameters

AN external neurostimulator (Nerve-On model, TODOC Co., Ltd, Seoul, Korea) was used for MFB electrical stimulation. The stimulation parameters were set as a biphasic current pulse with a 200-μs duration, 130 ± 5.16 μA amplitude over 300 ms at 230 Hz, as described previously [30].

### Intracranial self-stimulation (ICSS) operant conditioning

An operant conditioning chamber (Med Associates, model: ENV-008, Fairfax, VT, USA) with inner dimensions of 30.5 cm × 24.1 cm × 21.0 cm was used for operant conditioning. The ICSS training in the chamber started 3 days after surgery. Each rat was connected to an electrical stimulator via wire leads. The rats were allowed to freely explore the chamber, and when the rat pressed a lever, MFB electrical stimulation was provided. The rats were trained for 7 consecutive days until they reliably pressed the lever. Rats that pressed the lever > 40 times/min were used for the BMI task experiment.

### ECoG recording

To set up the system, the main controller was implemented using the Matlab software (MathWorks, Natick, Massachusetts, USA) for real-time control of the recording system and real-time signal processing. Two RHD 16-channel recording headstages (Part #C3334, Intan Technologies, LLC., Los Angeles, California, USA) were connected to each left and right recording site. Each headstage was connected by an ultra-thin SPI cable (1.8 m, Part #C3216, Intan Technologies, LLC., Part #C3334, Los Angeles, California, USA) to the neural signal recording system RHD USB Interface Board (Intan Technologies, LLC., Part #C3334, Los Angeles, California, USA). The acquired neural signal was sampled at a frequency of 10 kHz and bandpass filtered in the range of 0.1–250 Hz. ECoG data was downsampled to 500 Hz and a fast Fourier transform (FFT) was conducted every 300 ms. The RHD MATLAB toolbox and Matlab were used on a 64-bit Windows 7 operating system for the BMI task.

### BMI task learning/training session: controlling a visual object via ECoG signals

The BMI task learning session was started after ICSS operant conditioning. In order to let the animal familiarized to the environment for the BMI task, on the first day, the rat was allowed to freely explore the operant chamber where a monitor was positioned on its wider side. The recording cable was disconnected, and only the MFB stimulation cable was connected to the animal. On the 30.5 cm × 23.0 cm-sized monitor (LG, model name: L1930S, Seoul, South Korea), the background color was set to black and a white circular object (hereafter referred to as a dot) appeared on the left or right end of the monitor. The BMI task learning session was performed in a dark room to allow the animal to easily recognize the white dot. The diameter of the white dot was set to 8.7 cm. The dot moved on the x-axis in the range between − 31 and 31 steps. One step was equal to 5 mm. Considering the dot size, the initial position of the dot was randomly determined at either + 28 or − 28. During the learning session, the dot movement on the screen was shown to the animal according to predetermined scenario without actual neural recording. The dot moved within this range according to the scenarios; thus, when the next dot position fell out of this range, it was reset to ± 28 to prevent dot disappearance. Two vertical red lines were located to show the target zone on the screen center. When the animal showed interest in the monitor and watched the moving dot arriving to the target zone, the animal was rewarded with MFB stimulation. A single simulation of a dot movement took 27 s and total 100 simulations were shown to the animal. Among 100 simulations, 92 simulations were scenarios of a successful case providing MFB stimulation. In the other 8 scenarios, the dot was moving but did not arrive the target zone. During this learning session, the MFB stimulation was provided under supervision of the experimenter. Thus, if the animal was not watching the monitor nor focusing on the moving dot, the MFB stimulation was not provided.

The ECoG-based BMI task training session started on the next day and the implanted electrodes were connected to both the recording and stimulation systems. The training session consisted of 7 sessions and 10 trials per session. A signal trial was set to 1 min and total 10 trials were conducted on a single day. The dot was initially positioned randomly on the left or right end of the screen. Then the dot was moved based on the animal’s ECoG signals. The average power spectrum within a specific frequency band (40–70 Hz gamma band) was calculated from the recorded ECoG data in real time. The dot position was updated with the power spectrum at 300-ms intervals. In order to count as a success, the rat was required to hold the dot in the target zone for at least 600 ms. If they succeeded (in cases where 80% of the dot area was inside the target zone), they received MFB stimulation as a reward and this was counted as a success trial. If the rat failed for the trial, a new trial started immediately and the previous trial was recorded as a failed trial. In case the animal completed the task before the end of a trial, the dot was automatically reset to a new starting position on the left or right end of the screen. Since the animals can succeed multiple times during a single trial, we summated the number of success counts during 10 trials in a day and defined as “total success count”.

### Neural control of the dot positioning BMI task

We designed an online comparator to update the next dot position using the subject’s ECoG signal during the BMI task training session. The amplitudes of the power spectrums from left and right hemispheres were compared and the direction of dot movement was determined as the same direction of the hemisphere. The comparator updated the dot position at 300-ms intervals. The raw neural signal was recorded and saved to analyze brain activity during operation of the online comparator. The comparator algorithm used in the BMI task is described below:

$$\begin{aligned} & {\text{Displacement}} \\ & \quad = \left\{ {\begin{array}{*{20}l} {W_{right} } \hfill & {if\,({\text{Amplitude}}\,{\text{Spectrum}}_{{\left( {{\text{right}}\,{\text{hemisphere}}} \right)}} > {\text{Amplitude}}\,{\text{Spectrum}}_{{\left( {{\text{left}}\,{\text{hemisphere}}} \right)}} )} \hfill \\ { - W_{left} } \hfill & {if\,({\text{Amplitude}}\,{\text{Spectrum}}_{{\left( {{\text{right}}\,{\text{hemisphere}}} \right)}} < {\text{Amplitude}}\,{\text{Spectrum}}_{{\left( {{\text{left}}\,{\text{hemisphere}}} \right)}} )} \hfill \\ 0 \hfill & {if\,({\text{Amplitude}}\,{\text{Spectrum}}_{{\left( {{\text{right}}\,{\text{hemisphere}}} \right)}} = {\text{Amplitude}}\,{\text{Spectrum}}_{{\left( {{\text{left}}\,{\text{hemisphere}}} \right)}} )} \hfill \\ \end{array} } \right. \\ & \quad \quad \quad \quad \quad \quad ({\text{W}}_{{{\text{right}}}} \,{\text{and}}\,{\text{W}}_{{{\text{left}}}} :{\text{weight}}\,{\text{factor}}\,{\text{of}}\,{\text{each}}\,{\text{hemisphere}}) \\ & \quad \quad \quad \quad \quad \quad {\text{New position}} = {\text{previous}}\,{\text{position}} + {\text{Displacement}} \\ \end{aligned}$$

In the above formula, the amplitude spectrum was calculated from ECoG data for each left and right hemisphere. ECoG data were recorded every 300 ms on which FFT was conducted to calculate the amplitude spectrum. A gamma band of 40–70 Hz was selected for dot position updating. W_right_ and W_left_ were the weight values to balance the spectrum of the brain signal between the left and right hemispheres. Both W_right_ and W_left_ were set to + 1 as default, however, these coefficients were updated depending on the initial ECoG recording from individual animals. For example, when the amplitude spectrum of one hemisphere is dominant than that of the other hemisphere, the position of the dot was biased to one direction so quickly. To prevent this, the dominant side of W_(left or right)_ was reassigned to a smaller value than 1. In this case, we tested the BMI task for 5 trials per animal to reset the value before the main BMI task training trials. W_right_ and W_left_ ranged between 0.3 and 1. The comparator compared the amplitude spectrum of each left and right hemisphere and calculated a new dot displacement. For example, if the amplitude spectrum of the right barrel cortex is larger than that of the left barrel cortex, the displacement is + 1 (position on the x-axis, which is 5 mm to the right direction) as default.

### Statistical analysis

All graph data are presented as mean ± standard error of the mean (S.E.M) and were analyzed using GraphPad Prism 9.0 (Graph Pad Software, San Diego, USA) software. Data from six rats were used for statistical analysis. All behavioral training and testing data were analyzed via paired *t*-tests and one-way ANOVA. *p* values of < 0.05 were considered to indicate statistical significance. Post test for liner trend were used additionally to analyzed BMI task behavioral data.

## Results

**Fig. 3:**
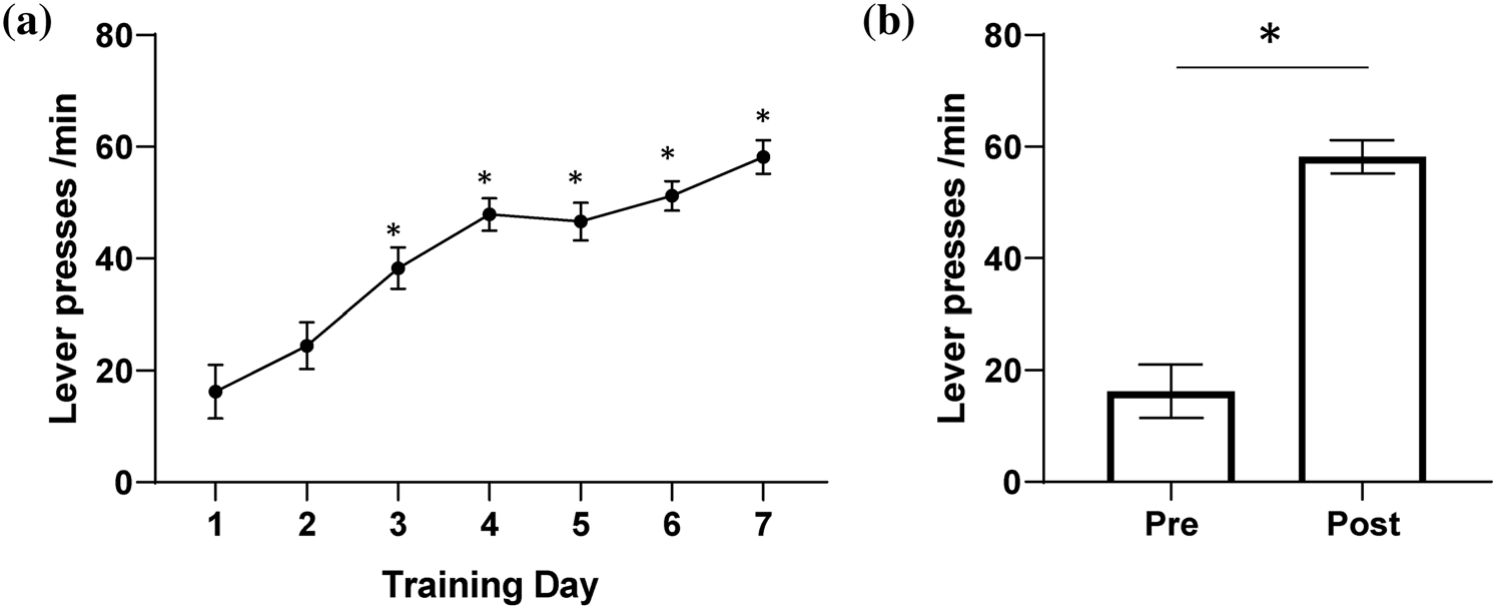
Intracranial self-stimulation (ICSS) **a** Lever presses per min by days (*p < 0.05). **b** Comparison of lever presses pre- and post-ICSS (*p < 0.05)

To establish a BMI rat model, ICSS operant conditioning was performed after a 3-day recovery to ensure that MFB stimulation provides virtual rewards for operant conditioning. Figure 3a, b show that the lever press rate changed over the study period. The number of lever presses increased significantly from day 3 to day 7 (One-way ANOVA, *p* < 0.05). The number of lever presses on day 1 was 16.3 ± 4.8 presses/min, whereas that on day 3–6 was 38.3 ± 3.7, 47.9 ± 2.9, 46.6 ± 3.4, and 51.2 ± 2.6 presses/min, respectively. After 7 days of ICSS, compared with day 1, the number of lever presses increased significantly to 58.2 ± 3.0 presses/min (paired *t*-test, *p* < 0.05). It was verified that the animals consistently pressed the lever > 40 times/min after day 4.

**Fig. 4:**
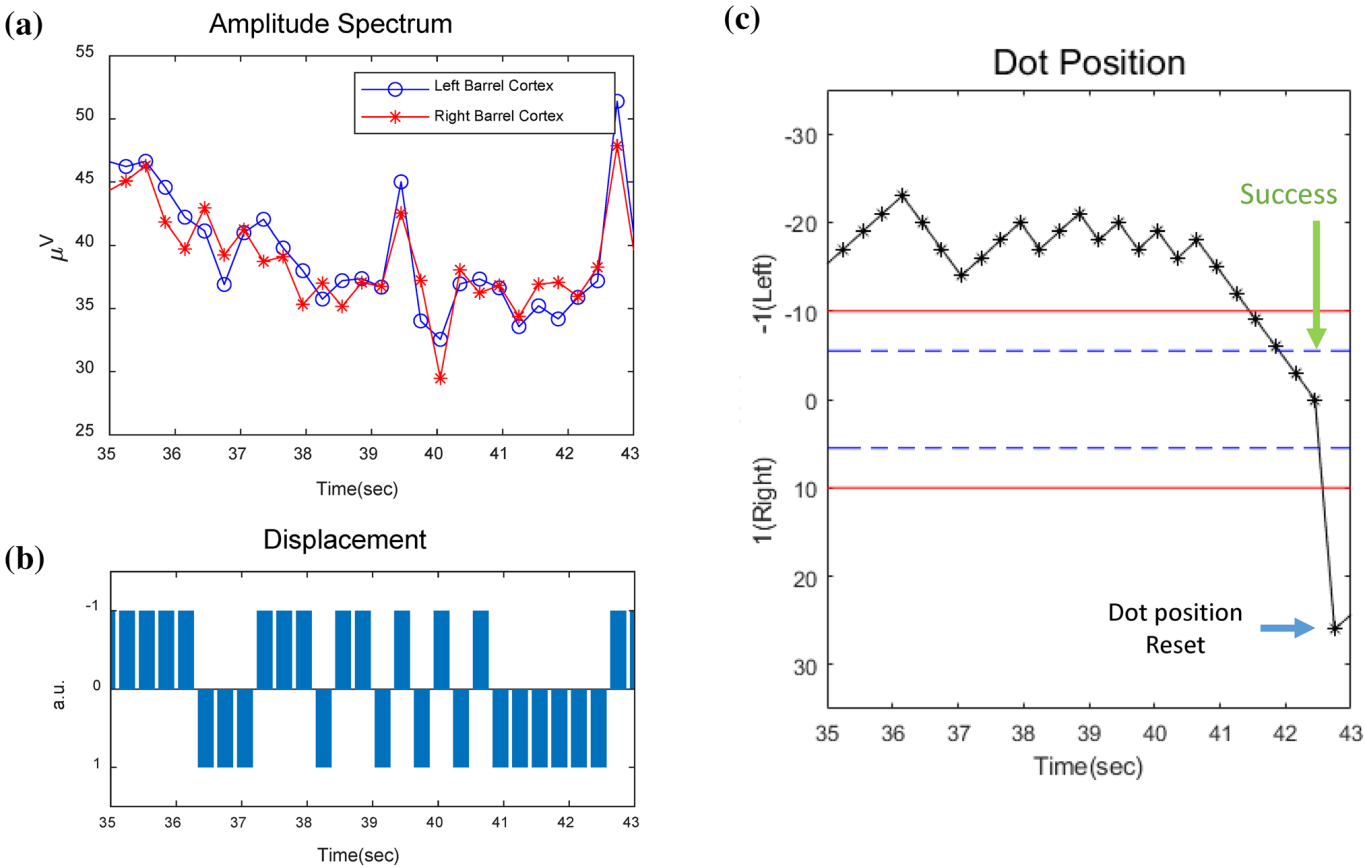
A sample of transformation of neural activity to dot position during a BMI task. **a** Amplitude spectrum between the gamma band (40–70 Hz) of the (blue) and right barrel cortex (red). **b** Converted dot displacement with direction. **c** Cumulative position of the dot on the screen

Figure 4 shows an example of real-time calculation for the proposed BMI operation. The amplitude spectrum was extracted from FFT at every 300 ms (Fig. 4a). The amplitude spectrum of each gamma band (40–70 Hz) was compared and the higher value was converted to the dot displacement value (Fig. 4b). For example, if the amplitude spectrum of the left barrel cortex is larger than that of the right barrel cortex, the dot moves to the left with the W value from the current position. While the animal watched the dot movement on a monitor, it tried to move the dot to the screen center to receive the reward. When the dot arrived to the targeted area and stayed more than 600 ms, the MFB stimulation was provided. Afterwards, the dot appeared on the left or right end of the screen randomly during the trial. When the animal accomplished the task before finishing the trial, the dot was automatically reset to a new starting position randomly on either left or right end of the screen (Fig. 4c). As described in Fig. 4c, after the dot successfully arrived in the target zone (green arrow), the dot had been moved to right end of the monitor for a new start (blue arrow).

**Fig. 5:**
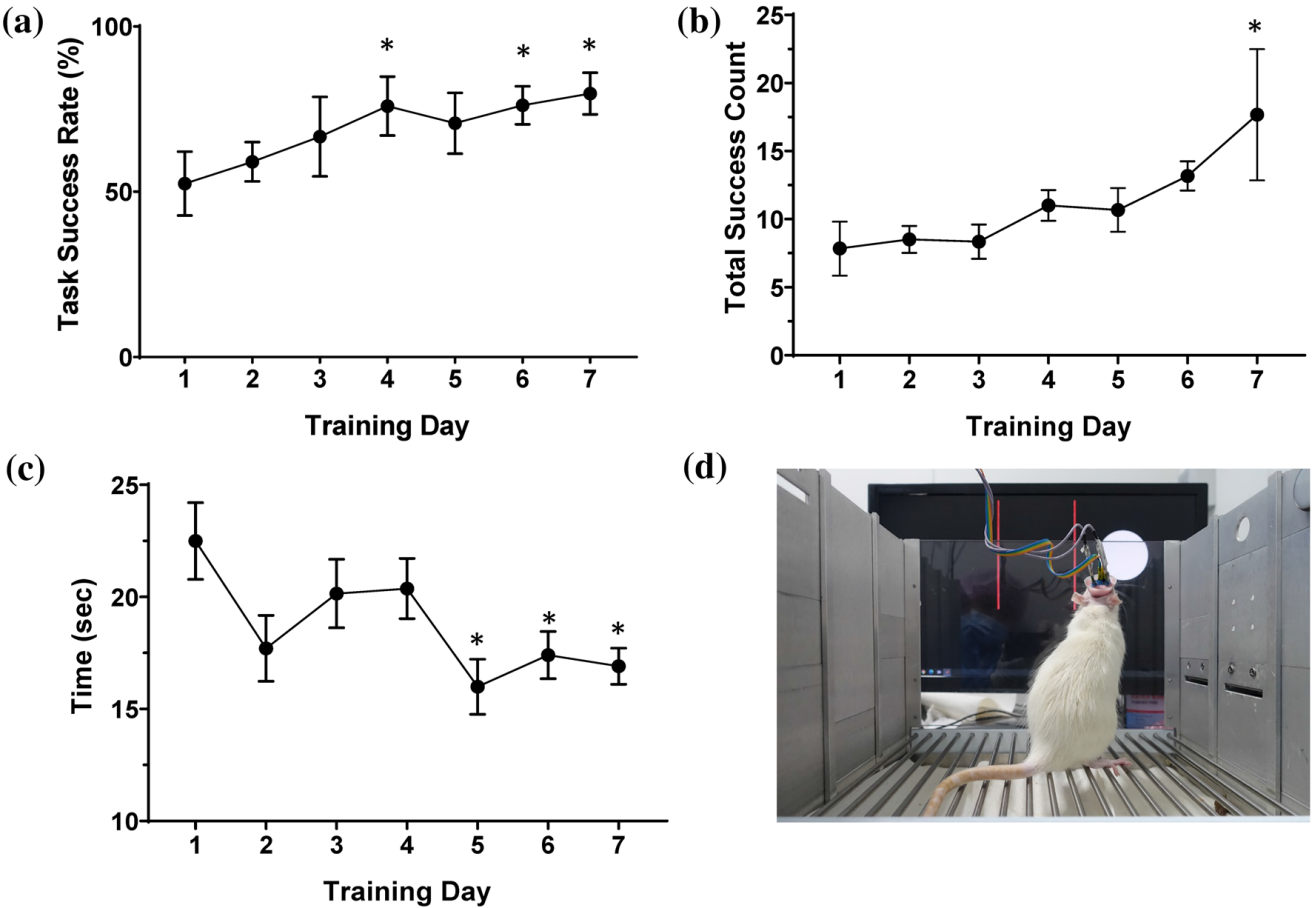
Operant conditioning BMI task. **a** Task success rate. (* for *t*-test with Day 1, *p* < 0.05). **b** Total Success Count. (* for *t*-test with Day 1, *p* < 0.05). **c** Time taken for a trial of the BMI task (* for *t*-test with Day 1, *p* < 0.05). **d** Rat in the ECoG-based BMI experiment environment

To get the rat to focus on the screen, a dot was moved continuously from side to side regardless of the rat’s neural signals during the learning session; when the rat saw the screen, the animal was administered with MFB stimulation to spike its interest in the moving dot (Fig. 5d). During the next 7 days of the training session, the animal learned to control the dot to earn the reward. The success rate showed a linear trend (linear trend test, R^2^ = 0.163, F = 7.05, *p* < 0.05; Fig. 5a). The success rate increased from 52.5 ± 9.7% to 79.7 ± 6.3% (paired *t*-test, *p* < 0.05). Total success counts also showed a linear trend (linear trend test, R^2^ = 0.246, F = 12.2, *p* < 0.05; Fig. 5b). Further, the time required to complete the task decreased significantly from day 5 onward (One-way ANOVA, F = 3.18, *p* < 0.05; Fig. 5c). On day 1, it took the animals 22.49 ± 1.7 s to move the dot to the target zone, whereas from day 5 to 7, it took 16.0 ± 1.2, 17.4 ± 1.1, and 16.9 ± 0.8 s, respectively.

**Fig. 6:**
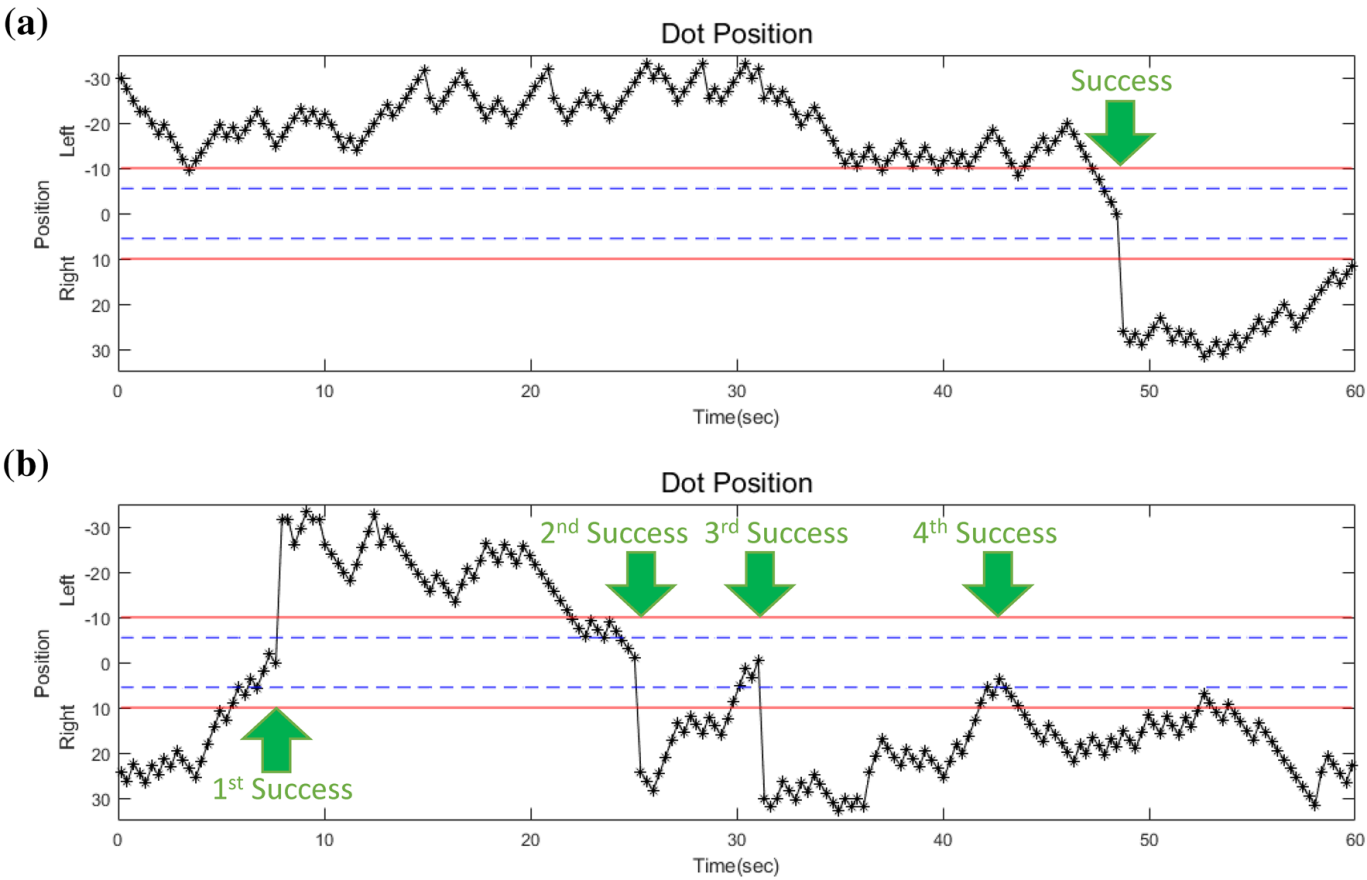
Examples of success trials. The two red lines indicate a target zone displayed on the monitor. The blue dash line indicates the threshold for success. **a** Single success in a single trial (day 2). **b** Multiple successes in a single trial (day 4)

To prevent the directional preference of the animal, the dot is located randomly on the right or left side at the starting time point of the trial. In all trials of the six rats performed during the 7-day session period, each dot started on the right and left side of the monitor 259 and 269 times, respectively. In addition, the success rates of the right and left starting trials were 57.14% and 55.76%, respectively. Figure 6 shows two examples of successful trials in the same animal. In the early training stage (day 1-3), a single success was counted (Fig. 6a). Otherwise, in the late training stage (day 4-7), multiple successes were observed in a single trial (Fig. 6b). No significant differences or tendency was observed in daily success counts or the proportion of single and multiple success counts. (Fig. 6).

## Discussion

Since brain is a complex and intelligent organ that operates delicately with various inputs, the rats can learn the BMI task through operant conditioning. In this study, we established a simple BMI model for rats which included the interpretation of directional intention from whisker somatosensory cortex and the provision of virtual reward to the rats. To investigate whether the rats can be trained to utilize the BMI model to control the movement of the dot on the screen, the behavioral sessions were repeated. As shown in the result, the animal successfully controlled the dot’s movement towards the desired location using the spontaneous neural activity from whisker somatosensory cortex. It indicates that the whisker somatosensory cortex can an appropriate brain target region for BMIs from which the animal’s intention can be retrieved adaptively.

Generally, BMIs use neuronal action potentials (spikes) recorded by implanted multichannel depth-type microelectrode arrays [1–9, 15–17]. In general, this invasive method has a higher spatial resolution and a better quality of neural signals than the non-invasive method like scalp electroencephalography. However, spike-based BMIs have a critical limitation due to tissue response to the implanted electrode. The number of neurons recorded by implanted electrodes gradually decreases over months [33]. Owing to degradation and mechanical breaks, a biological foreign-body response to injury including reactive astrogliosis and microgliosis can progressively lead to scarring and neuronal death around the electrode [34]. Therefore, it is a big challenge to maintain long-term stable recordings of the same individual neurons.

To circumvent this problem, in this study, we utilized the ECoG signals instead of the spike signals to operate the BMI system. The extracellular potentials recorded by electrodes in cortical areas comprise multiple components in distinct frequency bands, which may contain movement-related information. Usually, broadband power at high frequencies (gamma: 30–150 Hz) is positively correlated with neuronal firing rates [35] and may reflect the summation of action potentials and synaptic currents associated with a desynchronized, strongly active neuronal population. Costecalde et al*.* demonstrated that a BMI task could be used to control food dispensers via the ECoG signals of freely moving rats; the system was operated for a year, a long period for a rat BMI model [36]. They also utilized gamma band (50-180 Hz) in the cortex to operate the system. In addition, Jung et al*.* compared the BMI learning performance of the alpha (8–13 Hz), beta (13–30 Hz), and gamma (30–55 Hz) bands of ECoG motor cortex signals in a rat model; the gamma band ECoG-based BMI showed the best performance among all frequency bands [18]. Based on these previous BMI studies with the gamma band neural signals, we determined to use the power spectrum in 40–70 Hz gamma band, which overlapped with the gamma band ranges in the literature.

In order to achieve the best performance of BMI systems, in principle, it is essential to understand how the brain decode the neural signals for specific motor functions. However, Lang et al. developed a simple encoding-based BMI animal model using neuronal activity in the PFC, based on the hypothesis that neural signals do not need to be fully decoded for BMI systems as the brain is a complex learning system where the animal can learn an arbitrary task with appropriate training [15]. In addition, Jung et al. applied a simple decoding algorithm to compare BMI learning performance between various frequency bands and brain areas [18]. These studies focused on training to enable a rat to control an external device by decoding the animal’s intention to move an external object. Animal training involves operant conditioning. Generally, food and water are restricted and used as a positive reward during behavioral sessions. However, this strategy should be carefully performed to obtain successful results from animal behavioral experiments. Deprivation requires careful and time-consuming monitoring of food or water consumption to avoid causing animal discomfort while maintaining their motivation. Moreover, the motivation levels are difficult to control depending on the animal; moreover, once the animal is satiated, they stop performing. Therefore, despite a well-established procedure, this operant conditioning with food and water has clears limitations. On the other hands, deep brain stimulation on MFB easily induces neural circuit reinforcement during behavior learning. Excitation inputs on the MFB, which connects the brain’s reward regions, induce strong motivation for activation. With optimized stimulation parameters, deep brain stimulation on the MFB does not require the time for food and water deprivation and allows the experiment to be conducted consistently regardless of the animal’s appetite.

The whiskers are a well-known highly developed sensing organ in rats; whisking is an essential behavior for survival. Therefore, many studies investigated whisker movements and the related brain areas and neurological connections [26]. Behavior-level studies revealed the relationship between head movement and active/passive whisker movements and right/left asymmetries of the movements. Each whisker has a one-to-one anatomical mapping in the whisker-related somatosensory cortex, also known as the barrel cortex. Incoming sensory information to the S1BF is sent to directly connected cortical and subcortical brain regions. In addition, the whisker-related somatosensory cortex receives input from higher-order parts of the thalamus and various neuromodulatory inputs such as acetylcholine, dopamine, and serotonin [26]. The synaptic networks of the S1BF contribute to sensory perception, learning, and motor output. Several brain areas are involved in the execution of simple goal-directed sensorimotor transformations. In the rat model, it is known that the frontal cortex, medial PFC, dorsal hippocampal area CA1, and striatum influence the S1BF [26]. During goal-directed learning, reinforcement could occur through the strengthening of certain neural circuits linking whisker-related to dot control-related parts of the brain. In this study, we used MFB excitation for the reinforcement because it releases dopamine which is the most prominent reward-related neurotransmitter in the brain. The artificial increase in the firing rate of dopaminergic neurons excites the striatum and PFC but not the S1BF. The striatum is supposed to imply an important role in action selection and initiation [26]. Moreover, striatal neurons receive thalamic input, and the cortex might play a key role in directing the appropriate plasticity of thalamostriatal synapses [26]. Because the S1BF receives input from the ventral posteromedial nucleus of the thalamus, the sensory cortex might enable cortical plasticity. In this study, it is hard to conclude which interaction mainly is involved to complete the neural circuit that projects the animal behavior to success this BMI task. To seek the direct/indirect pathway optogenetics tools would provide the information of an uncertain connectivity between somatosensory cortex and thalamus.

In summary, we implemented an ECoG-based BMI system in freely moving rats. They successfully learned to control a simple one-dimensional dot moving for the BMI task with gamma band ECoG signals of the somatosensory cortex. MFB stimulation was provided as a virtual reward to the rats once they accomplished the task. This reward motivated them to control the BMI task multiple times in the behavioral session. Repetitive training with powerful rewards may influence the cortical plasticity of the sensory cortex, which indicates that higher cognitive functions modulate the activity of the somatosensory cortex in a top–down manner. Therefore, our findings suggest that somatosensory ECoG signals have potential for future application in ECoG-based BMI technology and would allow neuronal control of unrestrained movements in people with neurological disorders or disabilities affecting motor-related brain areas.
